# Exploration of Piperidinols as Potential Antitubercular Agents

**DOI:** 10.3390/molecules191016274

**Published:** 2014-10-10

**Authors:** Areej Abuhammad, Elizabeth Fullam, Sanjib Bhakta, Angela J. Russell, Garrett M. Morris, Paul W. Finn, Edith Sim

**Affiliations:** 1 Department of Pharmacology, University of Oxford, Mansfield Road, Oxford OX1 3QT, UK; 2 Department of Pharmaceutical Sciences, Faculty of Pharmacy, University of Jordan, Queen Rania Street, Amman 11942, Jordan; 3 Chemistry Research Laboratory, Department of Chemistry, University of Oxford, Mansfield Road, Oxford OX1 3TA, UK; 4 School of Life Sciences, University of Warwick, Coventry CV4 7AL, UK; 5 InhibOx, Oxford Centre for Innovation, New Road, Oxford OX1 1BY, UK; 6 Faculty of Science, Engineering and Computing, Kingston University, Penrhyn Road, Kingston KT1 2EE, UK; 7 Department of Applied Computing, University of Buckingham, Hunter Street, Buckingham MK18 1EG, UK

**Keywords:** tuberculosis, covalent inhibitors, piperidinols, arylamine *N*-acetyltransferase

## Abstract

Novel drugs to treat tuberculosis are required and the identification of potential targets is important. Piperidinols have been identified as potential antimycobacterial agents (MIC < 5 μg/mL), which also inhibit mycobacterial arylamine *N*-acetyltransferase (NAT), an enzyme essential for mycobacterial survival inside macrophages. The NAT inhibition involves a prodrug-like mechanism in which activation leads to the formation of bioactive phenyl vinyl ketone (PVK). The PVK fragment selectively forms an adduct with the cysteine residue in the active site. Time dependent inhibition of the NAT enzyme from *Mycobacterium marinum* (*M. marinum*) demonstrates a covalent binding mechanism for all inhibitory piperidinol analogues. The structure activity relationship highlights the importance of halide substitution on the piperidinol benzene ring. The structures of the NAT enzymes from *M. marinum* and *M. tuberculosis*, although 74% identical, have different residues in their active site clefts and allow the effects of amino acid substitutions to be assessed in understanding inhibitory potency. In addition, we have used the piperidinol 3-dimensional shape and electrostatic properties to identify two additional distinct chemical scaffolds as inhibitors of NAT. While one of the scaffolds has anti-tubercular activity, both inhibit NAT but through a non-covalent mechanism.

## 1. Introduction

Although tuberculosis (TB) is treatable with well-established drugs, and even preventable through vaccination, the disease remains one of the leading causes of death by bacterial infection, being responsible for approximately two million deaths annually. Tuberculosis infects one-third of the human population. It is estimated that 8.6 million new infections occurred in 2012, and thus it affects more people today than at any other time in history [1,2]. Until very recently TB has not been in the research portfolios of most of the major pharmaceutical companies. This neglect is partly because the greatest burden of disease is in developing countries [2]. A significant proportion of TB cases and deaths occur in human immunodeficiency virus (HIV) positive people [2].

The current drug treatment is more than 40 years old, while the vaccine is almost 100 years old. Although the existing TB treatment is effective against drug-susceptible bacilli, the continuation of treatment for at least six months is essential in order to kill persistent or slow growing strains [3]. The emergence of multi-drug resistant (MDR) and extensively-drug resistant (XDR) TB further adds to the burden [4] and requires treatment with second-line drugs, which are less effective, more toxic, and expensive. Recently totally-drug-resistant TB (TDR) has been reported [5,6].

Bedaquiline (TMC-207), which inhibits mycobacterial ATP synthase, was approved by the FDA as part of the treatment regimen for MDR-TB [7,8]. Thus, it is the first new TB drug since the introduction of rifampin in 1970 [9].

Although the availability of the *M. tuberculosis* genome and the identification of potential targets are leading to filling of the drug pipeline with agents such as benzothiazinones [10], new targets need to be identified to avoid the problem of drug resistance in the future. The need for the physicochemical properties of potential compounds also needs to be addressed to allow penetration of the uniquely impermeable cell envelope, and to find an escape from active drug efflux mechanism in mycobacteria.

There is a history of the use of pro-drugs as anti-mycobacterials. Isoniazid, the front-line anti-tubercular, is a pro-drug, which is activated within the bacterium [11] and acts through a covalent binding mechanism within *M. tuberculosis*. Traditionally, medicinal chemists have been skeptical about covalent drugs because of concern regarding potential for off-target reactivity. However, there has recently been a resurgence of interest in covalent drugs, with their potential advantages being increasingly recognized [12].

**Figure 1:**
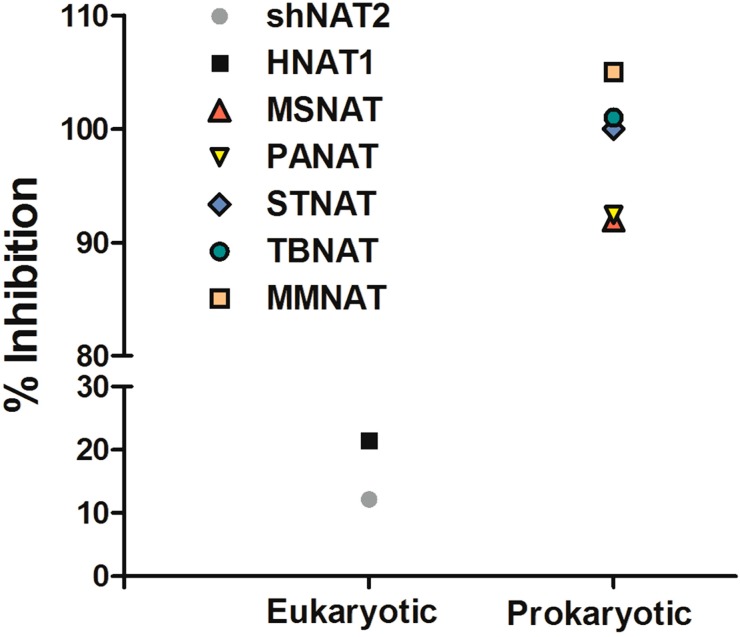
Specificity of **1** for prokaryotic *N*-acetyltransferase (NAT) enzymes.

Compound **1**, a piperidinol derivative, has been identified as a potent antimycobacterial (MIC < 5 μg/mL) which also inhibits mycobacterial arylamine *N*-acetyltransferase (NAT) (Figure 1) through a covalent mechanism of action [13]. The NAT enzyme in mycobacteria has been identified in several studies as a potential target for the treatment of tuberculosis in that the gene is part of a cluster essential for *M. tuberculosis* survival inside macrophage [14,15,16]. MMNAT (NAT enzyme from *Mycobacterium marinum*), at 74% identical has been exploited as a model for *M. tuberculosis* NAT (TBNAT) since the MMNAT enzyme is highly soluble and a crystal structure has been available for some time. Although recently a preparation of TBNAT and a crystal structure have been available, the protein from *M. tuberculosis* is much more difficult to handle and particularly in relation to obtaining the high concentrations which best support crystallization.

Compound **1** has previously been investigated as a candidate for several therapeutic uses, including anti-inflammatory [17], anticancer [18], anticonvulsant [19], antimicrobial [20] and historically as an antitubercular [21]. Furthermore, compound **1** is a cyclic derivative of a Mannich-base that has been investigated as a prodrug vehicle for several therapeutic agents [22]. Understanding the key features of this scaffold is essential in progressing the development of the compound as a therapeutic agent.

Compound **1** was tested at 30 μM against five pure recombinant NAT enzymes from *M. smegmatis* (MSNAT), *S. typhimurium* (STNAT), *P. aeruginosa* (PANAT) and also against two eukaryotic enzymes, hamster NAT2 (shNAT2) and human NAT1. The NAT activity assay [23] was used to determine the specific activities of the pure recombinant NATs in the presence of compound **1**. The results are shown as the mean ± S.D. of triplicate determinations of percentage inhibition of hydrolysis of Ac-CoA as described in the presence of 500 μM 5-amino salicylic acid (5ASA) as substrates for all enzymes apart from TBNAT where 500 μM hydralazine (HLZ) has been used as a substrate. The inhibition is represented as a percentage compared to an uninhibited control.

To this end, we report the evaluation of chemical modifications on the piperidinol scaffold using inhibition of mycobacterial NAT by compound **1** and its analogues in order to try to improve potency. We have also carried out *in silico* studies with the piperidinol scaffold as a query molecule to open up chemical space on the basis of 3D shape and electrostatics and have identified two new chemical scaffolds, which were subsequently found to be NAT inhibitors. We have investigated the compounds as inhibitors of mycobacterial growth.

## 2. Results and Discussion

### 2.1. Mechanism of Inhibition

**Figure 2:**
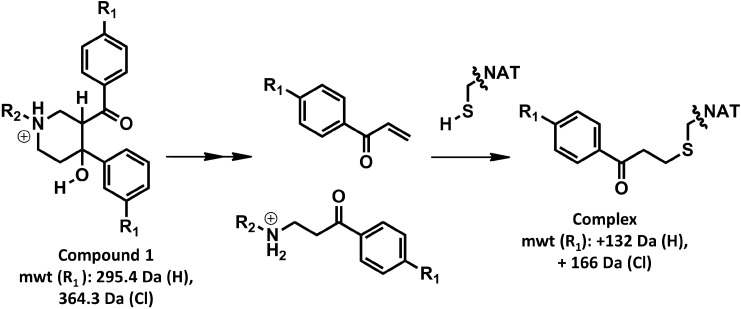
The mechanism of adduct formation.

Compound **1** has been identified as a selective inhibitor for bacterial and mycobacterial NATs [24,25]. The mechanism of NAT inhibition by this compound and its analogues has been elucidated and involves specific and unique covalent modification of the active site cysteine of NAT [13] (Figure 2).

The adduct formation is proceeded by the formation of a reactive phenyl vinyl ketone (PVK). The PVK reaction with thiols resulted in the addition of a 3-Phenyl-3-oxopropyl moiety (POP) (when R_1_ is H) or a 3-(4-Chlorophenyl)-3-oxopropyl moiety (when R_1_ is Cl). The expected differences in molecular weight of the added fragments are +132.07 Da and +166 Da, respectively.

The NAT enzyme from *M. marinum* is 74% identical to NAT from *M. tuberculosis* and the *M. marinum* NAT enzyme has been studied as a model for the less stable *M. tuberculosis* enzyme [13,26,27].

Using a selected set of compound **1** analogues (Table 1), the inhibition was demonstrated to be irreversible and time-dependent using the recombinant NAT enzyme from *M. marinum*, MMNAT, as has previously been described for inhibition of NAT from *M. tuberculosis* [13] (Table 1 and Figure S1 in Supplementary Materials). To facilitate comparison of the compounds, the values of the apparent inactivation half-life of the enzyme were calculated in the presence of the different compounds from *k_obs_* as shown in Table 1. Potent inhibitors are predicted to exhibit a shorter inactivation half-life [28]. An adduct is formed, in which a phenyl vinyl ketone moiety (PVK) is directly conjugated to the active site cysteine sulfhydryl group [13]. For compounds **2**, **3** and **5**, the structure of the inhibitor has a halide substituent on the *para* position of the benzene ring (position R_1_, Table 1), and thus the adduct formed with the active site cysteine is predicted to be larger than the benzene ring unsubstituted at the *para* position, as is observed by mass spectroscopy analysis [13].

The piperidinols exhibited the same type of inhibition against the homologue of NAT from *M. tuberculosis*, TBNAT, which is 74% identical in sequence to MMNAT and shares the key features of the binding pocket. Whilst the inhibition was more rapid with TBNAT than with MMNAT, higher concentrations of inhibitor were required compared to those used with MMNAT. The mechanism of activation of the compounds have been described in Abuhammad *et al.*, 2012 and is proposed to procede by a nucleophilic attack on the piperidinol ring carbonyl. We have not been able to ascertain whether the activation is enzyme catalyzed or is due to hydrolysis within the binding pocket. However, it is likely that the binding of the compounds into the active site promote the formation of the active intermediate since only the active site Cys is modified. If the active species were formed through hydrolysis without binding to the enzyme a priori, this would be likely to result in a random modification of all of the cysteine residues.

**Table 1:** The time-dependent activity of the piperidinols against MMNAT. 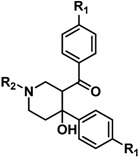

Code	R_1_	R_2_	*k_obs_* (10^−3^ min^−1^)	t_1/2_ (min)	Critical Volume (cm^3^/mol)	cLogP
1	H	-CH_3_	9 ± 2	81.5	864.5	2.41
2	Cl	-CH_3_	110 ± 2	6.3	962.5	3.53
3	Br	-CH_3_	74 ± 7	9.4	988.5	4.07
4	H	-CH_2_CH_3_	15 ± 1	45.6	920.5	2.75
5	F	-CH_2_CH_3_	638 ± 120	1.1	956.5	3.07
6	H	-(CH_2_)_3_CH_3_	104 ± 8	6.6	1032.5	3.66
7	H	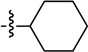	573 ± 25	1.2	1077.5	3.96
8	H	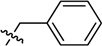	10 ± 1	71.4	1092.5	4.15
9	H	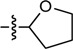	163 ± 39	4.2	994.5	2.84
10	H	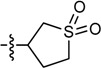	19 ± 1	37.1	1017.5	0.91
11	H	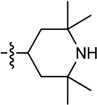	34 ± 1	20.2	1276.5	3.06

The assay was performed as described in Methods and *k_obs_* values were obtained from the slope of the semi-logarithmic plots of the residual activity *vs.* incubation time at 11.9 μM except for **5**, **7** and **11** (5.9 μM) and 10 (23.8 μM). The results are presented as the mean ± S.D. of triplicate measurements at 24 °C. t_1/2_ is the apparent inactivation half-life calculated from *k_obs_* (t_1/2_ = 0.693/k_obs_). The critical volumes (the volume of one mole of material at the critical temperature and pressure) and cLogP values (the octanol-water partition constant) as predicted by ChemBioDraw Ultra 12.0 [29] are shown. Compounds **1**, **2**, **7** and **11** have been described previously [13] and are included for full comparison.

### 2.2. Effects of Substitutions on the Piperidinol Scaffold

When the phenyl ring was *para* substituted with a halide, the resulting piperidinols (**2**, **3** and **5)** displayed an increase in the inactivation potency as evidenced by the shorter inactivation half time (Table 1) than the benzene ring unsubstituted at the *para* position. The fluoro derivative (**5**) showed a low inactivation time (Table 1). The ethyl substituent on the nitrogen atom in **5** is likely to contribute to the increased inactivation potency since the ethyl piperidinol **4** with no halide substituent, shows twice the activity of the methyl derivative **1**. However, other factors, for example the chemical stability of the compounds, could well contribute to their relative protein inactivation activities.

Increasing the length of the alkyl chain on the piperidinol nitrogen results in increased inhibition (as measured by the rate; Table 1; compounds **1**, **4** and **6**). However, this is unlikely to be due to hydrophobicity alone, because there is a significant difference in activity between the potent cyclohexyl analogue, compound **7**, and the weak benzyl analogue, compound **8**, despite both compounds having similar clogP values (Table 1, Figure S2 in Supplementary Materials). The cyclohexyl ring has a strong preference to adopt a chair conformation whilst the ring of the phenyl is planar. Furthermore, the extra methylene in the benzyl group inserts two rotatable bonds not present in the cyclohexyl compound, making it more conformationally flexible, as well as significantly altering the geometry of the attachment. Either of these points could contribute to reduced activity of the benzyl compound despite increased bulk.

Although there is no simple correlation with hydrophobicity, it appears to be important since highly polar R_2_ substituents result in poor inhibition of MMNAT. In addition to hydrophobicity factors, the volume of the inhibitors appears to be a contributing factor to inhibitory potency (Table 1). Except for compounds **3**, **7** and **10**, the inhibitor potency was found to increase linearly as the critical molar volume of the molecule increased (Table 1).

### 2.3. Comparison of MMNAT and TBNAT

TBNAT and MMNAT, although being 74% identical in sequence, show different substrate and inhibition profiles [30]. To provide a basis for a qualitative comparison of MMNAT and TBNAT inhibition by the piperindols, IC_50_ analyses were carried out. The IC_50_ values were comparable for inhibitors **1**–**6** and **11**; however, there is a difference between the activities of inhibitors **8**–**10** when tested against MMNAT compared with TBNAT (Table 2).

Evidence for binding of the piperdinols in close proximity to the methionine moieties Met209 and Met222 of MMNAT can be deduced from structural studies on the MMNAT complexed with compound **1** [31]. The refinement the crystal structure of MMNAT soaked with compound **1**, shows patches of electron density, contiguous with the methionine residues (Met209 and Met222) and the tryptophan Trp97, indicating the presence of a bound chemical entity(ies). The aromatic rings and the methylamine moiety of **1** have been modelled into these electron density patches (hypothetical model, Figure S3, Supplementary Materials). Notably, this electron density was absent in all other MMNAT structures (PDB entries 2vfb, 2vfc [32]; and 3ltw [27]), excluding the possibility that it was due to components from the expression system or from the crystallization cocktail as has been observed in e.g., NAT from *Bacillus anthracis* [33]*.* The interpretation of the electron density is proposed to be caused by the presence of the ligand in the enzyme’s active site before activation into the reactive intermediates. It is proposed that once the piperidinol is activated to the PVK fragment (Figure 2) the fragment is likely to react immediately with the active site cysteine. It is possible that the piperidinol fragment may be activated by the enzyme acting as a catalyst despite the relatively acidic pH (6.5) at which the crystals grew, as the enzyme was shown to maintain a stable catalytic (acetyl-transfer) activity within a pH range of 6–9. However, further evaluation of the pH stability of the piperidinols would need to be pursued for drug design.

**Figure 3:**
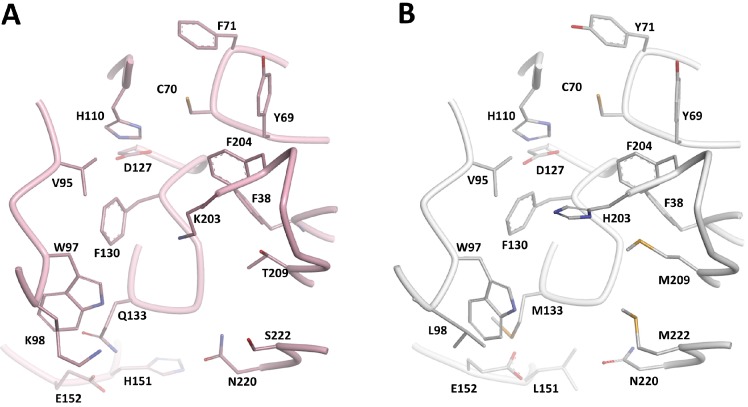
Comparison of the binding pockets of TBNAT and MMNAT. A ribbon representation shows the main residues in the binding pocket of (**A**) TBNAT and (PDB code 4BGF, 2.1 Å) and (**B**) MMNAT (PDB code 2VFB; 2.1 Å).

Therefore, we hypothesize that the piperidinols bind at this location within the MMNAT binding pocket. This region within the binding pocket varies significantly between MMNAT and TBNAT [34] (Figure 3) and is likely to explain the difference in the inactivation rate of these inhibitors on both enzymes (Table 2). The binding pocket of the TBNAT appears to be more charged and polar, compared to that of MMNAT [34]. The two methionine residues (Met209 and Met222) within this region of the binding pocket in MMNAT are substituted with tyrosine and serine in TBNAT, whilst the two leucine residues (Leu98 and Leu151) and methionine (Met133) are substituted with lysine, histidine and glutamine, respectively (Figure 3). This architecture is proposed to explain the reduced affinity of the piperidinols for TBNAT as evidenced by the need for higher concentrations to inhibit the TBNAT, although the rate of inactivation with TBNAT is faster than with MMNAT.

### 2.4. Effect on Mycobacteria

The compounds were also assessed for *in vitro* anti-mycobacterial activity against *M. bovis* BCG and the *M. tuberculosis* strain H37Rv (Table 2). All nine compounds showed promising antimycobacterial activity against *M. tuberculosis*, with an MIC below 10 μg/mL (Table 2).

In the absence of experimental evidence on comparison of uptake into *M. tuberculosis* and *M. bovis* BCG it is not possible to speculate on the reason for the minor differences. Nevertheless, the fact that these compounds do have activity in inhibiting growth identifies that they are of interest.

**Table 2:** The inhibitory activity of compound **1** and its analogues ^a^. 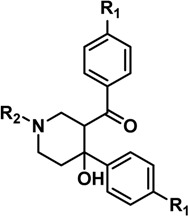

Code	R_1_	R_2_	TBNAT				MMNAT							MIC (μg/mL)			
			% Inhibition		IC_50_ (μM) HLZ		% Inhibition		IC_50_ (μM) HLZ			IC_50_ (μM) 5ASA		*M. bovis* BCG		*M. tuberculosis*	
1	H	-CH_3_	101 ± 1		7.7 ± 0.9		105 ± 1		1.3 ± 0.0			6.0 ±1		6.3–12.5		1–10	
2	Cl	-CH_3_	98 ± 1		1.6 ± 0.1		103 ± 2		0.16 ± 0.01			1.4 ± 0.6		6.3–12.5		ND	
3	Br	-CH_3_	98 ± 3		2.9 ± 0.4		99 ± 1		0.08 ± 0.01			1.3 ± 0.4		6.3–12.5		5–10	
4	H	-CH_2_CH_3_	72 ± 4		7.3 ± 0.3		126 ± 5		1.9 ± 0.0			8.0 ± 1		6.3–12.5		5–10	
5	F	-CH_2_CH_3_	108 ± 1		4.4 ± 0.0		102 ± 3		0.5 ± 0.0			1.3 ± 0.5		6.3–12.5		5–10	
6	H	-(CH_2_)_3_CH_3_	100 ± 3		6.9 ± 0.4		102 ± 3		2.6 ± 1			5.0 ± 0.4		6.3–12.5		1–5	
7	H	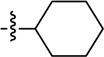	72 ± 60		4.4 ± 0.1		103 ± 1		ND			1.7 ± 0.2		6.3–12.5		1–5	
8	H	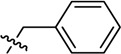	58 ± 2		ND		101 ± 1		4.1 ± 0.4			ND		6.3–12.5		0–1	
9	H	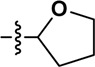	51 ± 3		ND		100.8 ± 0.5		2.5 ± 0.3			9.0 ± 0.9		ND		0–1	
10	H	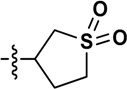	47 ± 2		ND		99 ± 0.7		13 ± 1			>30		3.1–6.3		ND	
11	H	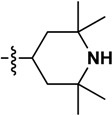	67 ± 4		1.1 ± 0.3		100 ± 2		2.7 ± 0.4			1.1 ± 0.3		6.3–12.5		1–5	

^a^ NAT activity was measured by a NAT-inhibition assay using 150 μM HLZ and 120 μM Ac-CoA as substrate. The percentage of enzyme inhibition was measured in the presence of 50 μM inhibitor and compared to the un-inhibited control. The IC_50_ values were determined by measuring the enzyme activity in the presence of variable concentrations of each inhibitor (0–250 μM) and compared to the un-inhibited control. The results are presented as the mean ± S.D. of triplicate measurements. ND is not determined. Inhibition curves were obtained by non-linear fitting of the% inhibition and the inhibitor concentration (μM) using the Log(inhibitor) *vs.* response module of GraphPad Prism 5.0.

### 2.5. In Silico Screening

**Figure 4:**
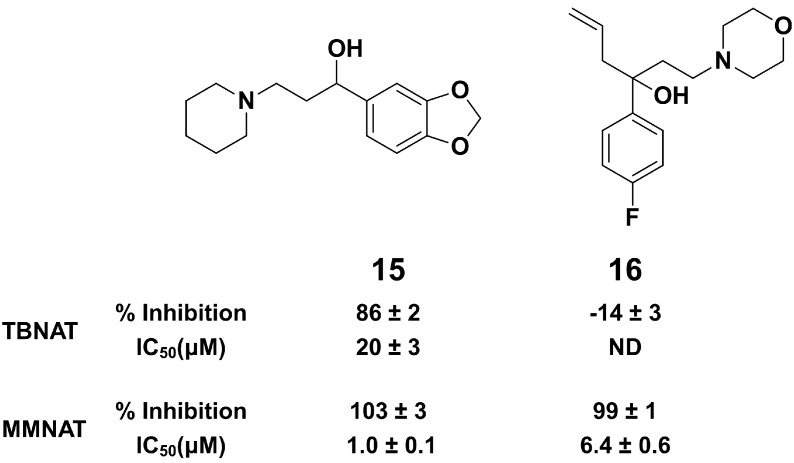
Active hits obtained by *in silico* 3D-shape screening.

The piperidinols are chiral molecules and the identification of other active chemotypes is desirable. Previously, ligand-based virtual screening for NAT inhibitors based on the 3D shape of compound **1** has proven very useful for identifying inhibitors of a distinct NAT homologue in humans [35]. Since then, a new methodology (ElectroShape) has been developed which includes an electrostatic comparison in the molecular similarity description in addition to shape [36,37]. Importantly, given the chiral nature of the query molecule, the molecular representation used by ElectroShape is sensitive to chirality, which was not the case in the previous work [38]. Screening for novel NAT inhibitors based on the 3D shape and electrostatic comparison of compound **1** was performed using the ElectroShape approach. A conformational model was generated for compound **1** after which it was used as a search query against a database of 7.3 million commercially-available molecules, Scopius (InhibOx Ltd., Oxford, UK). The identified hits were ranked based on the ElectroShape score and 12 compounds out of the top 100 hits were purchased and tested for their NAT inhibition activity. The chemical structures of the tested molecules and their inhibition activities are shown in Table S1 in Supplementary Materiala. Among these inhibitors, compounds **15** and **16** show NAT inhibition activities (Figure 4 and Figure S4 in Supplementary Materials). Although both **15** and **16** show high potency against MMNAT, only compound **15** shows inhibition of TBNAT.

The ability to identify inhibitors based on the 3D shape and electronic properties of compound **1** supports the affinity of the intact piperidinols to the NAT binding pocket substantiates, but does not prove, that the binding of the compounds into the active site promote the formation of the active intermediate. Although the search query molecule **1** acts via covalent bond formation, the hits obtained show no covalent adduct formation as was confirmed by mass spectroscopy analysis (data are not shown). Interestingly, compound **15** shows also antimycobacterial activity with MIC of 125–250 μM (33–66 µg/mL) against *M. bovis* BCG (Figure S5 in Supplementary Material).

## 3. Experimental Section

All chemicals and reagents were purchased from Sigma Aldrich (Poole, Dorset, UK), unless otherwise stated. Data analysis was carried out using GraphPad Prism 5.0. The critical volumes and cLogP values were predicted by ChemBioDraw Ultra 12.0 [29]. Structural figures and graphical renderings were made with Discovery Studio (DS) Visualizer 3.1 [39].

### 3.1. Range of Inhibitors

**Scheme 1:**
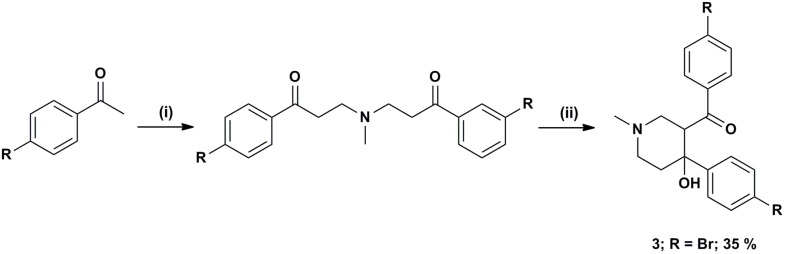
*Reagents and conditions*: (i) MeNH_2_·HCl, paraformaldehyde, cat. ZnCl_2_, MeCN, Δ, 16 h; (ii) NaOH.

Compound **1** which was identified from a previous high throughput screen was synthesized *ab initio* to confirm its identity and activities as previously described [40]. It has been confirmed separately that during the cyclisation only the diastereoisomer **1** depicted in Scheme 1 was formed [40]. The compound was obtained in 79% yield, with greater than 99.5% purity as determined by reverse phase HPLC.

Halogenated analogues **2** and **3** were either purchased from Chembridge, (San Diego, CA, USA) (compound **2)** or synthesized by cyclisation of the intermediate bis-Mannich bases with NaOH, which following recrystallization of the products furnished the corresponding piperidinols (compound **3**; Scheme 1).

To investigate further the structural influence of *N* functionality over both NAT inhibition and antimycobacterial activity, eight commercially available compounds (**4**–**11**, Cheshire Biosciences, Chester, UK) with different substitutions at the piperidinol nitrogen were selected for testing. All compounds were the best available grade. All compounds were greater than 95% pure apart from compound **4**, which was only 80% pure. Stock solutions of the all compounds were prepared at 5–50 mM in DMSO and stored at −20 °C.

### 3.2. Enzyme Production

The NAT enzymes from *M. smegmatis* [41], *S. typhimurium* [42], *P. aeruginosa* [43], *M. marinum* [32], *M. tuberculosis* [44], hamster NAT2 [45] and human NAT1 [46] were produced as recombinant proteins, purified as previously described and stored at −80 in 20 mM Tris-HCl pH 8 containing 1 mM dithiothreitol and 5% glycerol and thawed and used within 1–5 h.

### 3.3. NAT Inhibition Assay

An assay for measuring the formation of CoA [23] was modified and used to determine the activity of the enzyme in the presence of potential inhibitors exactly as described previously [13]. Irreversible inhibition, progressive with time was measured for the piperidinol inhibitors by the protocol described in [13] and the values of the apparent first order rate constant (*k_obs_*) were determined using the Kitz and Wilson model [28]. Adduct formation was determined as previously described by mass spectroscopy analysis [13].

### 3.4. Mycobacterial Growth Inhibition in Vitro

Mycobacterial growth inhibition was measured in one of two ways: on agar and in liquid culture.

Mycobacteria (*M. bovis* BCG and *M. tuberculosis* H37Rv) were grown as spot cultures in 6-well plates on solid medium (Middlebrook 7H10 medium supplemented with 10% (v/v) oleic acid-albumin-dextrosecatalase (OADC)) as previously described [14,47], with test compounds at the concentrations indicated in the text. Test compounds were added to the melted, partially cooled 7H10-OADC agar medium as solutions in DMSO, and the final concentration of DMSO in each well was 0.1% (v/v). The minimum inhibitory concentration (MIC) is defined as the concentration of inhibitor at which no growth of mycobacteria was detected after a period of 2 weeks. Compound **1** and its analogues that showed MIC below 5 μg/mL (**6**–**9** and **11**; Table 2) were tested *in vitro* for their cytotoxic effect on RAW 264.7 cells as previously described [48] and no cytotoxicity was observed at up to 50 μg/mL inhibitor concentration.

### 3.5. Alamar Blue Assay

The Alamar blue assay was used to determine the antimycobacterial activity of NAT inhibitors against *M. bovis* BCG str. Pasteur (ATCC 35734) [49,50].

*M. bovis* BCG (100 mL) was grown until early log-phase (OD_600_ 0.4–0.7). The cells were harvested (2500 g, 15 min), resuspended (50 mL phosphate-buffered saline (PBS), 0.05% Tween 80) and harvested (25,000 g, 15 min). The cells were resuspended (4 mL PBS, 0.05% Tween 80), aliquoted (100 μL) per cryo-tube and snap frozen in liquid nitrogen and stored at −80 °C. 7H9GC-OADC (2.4 mL) was added to 100 μL of cells and diluted 100-fold in 7H9GC-OADC. The assay was carried out in 96-well plates in the presence of either compound **4**, and INH as control (0–66 μg/mL in DMSO). Plates were incubated at 37 °C in the case of *M. bovis* BCG. Alamar Blue dye (50 μL) was added after six days for *M. bovis* BCG. The minimum inhibitory constant (MIC) was determined visually at the concentration at which color-change occurred (blue to pink).

### 3.6. In Silico Shape-Based Screening

*In silico* screening for potential NAT inhibitors was performed using the ElectroShape approach [37]. ElectroShape requires as input a three-dimensional representation of the query molecule, compound **1**. Because of the uncertainties around the specific bioactive conformation, a conformational model was constructed which consists of a representative set of low-energy conformations. Conformations were generated using the program Conform [51] keeping conformations within 10 kcal.mole of the global minimum and with a minimum RMSD between them of 0.5 Å. This conformational model was used as the query to screen the Scopius database of commercially available molecules (InhibOx Ltd., Oxford, UK). Scopius represents a consolidation of commercially available compounds from over 200 vendors worldwide. Only the drug-like compounds in Scopius, 7.3 million, were included in the virtual screening study. Drug-like compounds comply with Lipinski-like physicochemical properties and do not contain substructures (represented as SMARTS patterns) known to be problematic for lead optimization. The identified hits were ranked based on the ElecroShape score and 12 compounds of the top 100 hits were purchased and tested for their NAT inhibition activity.

## 4. Conclusions

The piperidinol scaffold was identified as a selective prokaryotic NAT inhibitor that exhibits potent antimycobacterial activity. The mechanism of inhibition of the NAT enzymes was confirmed previously by mass spectroscopy analysis and X-ray 3D-structure determination of the MMNAT enzyme with compound **1** [13]. The mechanism of NAT inhibition, which combines activation followed by covalent modification, presents an attractive starting point for novel anti-tubercular agents. Whilst NAT has been shown to be essential for intracellular survival of mycobacteria, it is likely that the piperidinols inhibit mycobacterial growth via inactivation of other targets also. Considering both the concept of polypharmacy and the resistance problems associated with TB treatment, more than one target is highly desirable.

In an attempt to improve antimycobacterial action and inhibition of NAT enzymic activity, we have studied a series of analogues with selective structural modifications. We have tested antimycobacterial actions on *M. bovis* BCG and *M. tuberculosis* and have used pure recombinant NAT enzymes from *M. marinum* and *M. tuberculosis*. The halogenation of the *para* position of the benzene rings as well as a hydrophobic *N* functionality showed improvement of the inhibition of MMNAT. The library of piperidinols tested exhibit different activity against MMNAT and TBNAT, despite the high sequence similarity between both enzymes. This is likely to be associated with the subtle differences in the architecture of the binding pocket of MMNAT and TBNAT.

Two new scaffolds of NAT inhibitors have been identified *in silico* using the 3D-shape and electrostatic modeling of compound **1** as a query molecule. The results demonstrate that the intact piperidinols have structural complementarity to the binding pocket of MMNAT such that non-specific affinity to the enzyme is excluded. Compound **15** identified through shape comparison was also demonstrated to inhibit the growth of *M. bovis* BCG.

The results clearly highlight the importance of the piperidinol scaffold as a lead in antimicrobial drugs development and underline the benefits of extending the number of new chemical entities through *in silico* methods.

## Supplementary Materials

Supplementary materials can be accessed at: http://www.mdpi.com/1420-3049/19/10/16274/s1.

## Supplementary Files

Supplementary File 1

## References

[1] Dye C., Williams B.G. (2010). The population dynamics and control of tuberculosis. Science.

[2] WHO Global Tuberculosis Report 2013. http://www.who.int/tb/publications/global_report/gtbr13_executive_summary.pdf?ua=1.

[3] Sarkar S., Suresh M.R. (2011). An overview of tuberculosis chemotherapy—A literature review. J. Pharm. Pharm. Sci..

[4] Shenoi S., Friedland G. (2009). Extensively drug-resistant tuberculosis: A new face to an old pathogen. Annu. Rev. Med..

[5] Udwadia Z.F., Amale R.A., Ajbani K.K., Rodrigues C. (2012). Totally drug-resistant tuberculosis in India. Clin. Infect. Dis..

[6] Rowland K. (2012). Totally drug-resistant TB emerges in India: Discovery of a deadly form of TB highlights crisis of “mismanagement”. Nat. News.

[7] Andries K., Verhasselt P., Guillemont J., Gohlmann H.W., Neefs J.M., Winkler H., van Gestel J., Timmerman P., Zhu M., Lee E. (2005). A diarylquinoline drug active on the ATP synthase of *Mycobacterium tuberculosis*. Science.

[8] Diacon A.H., Pym A., Grobusch M., Patientia R., Rustomjee R., Page-Shipp L., Pistorius C., Krause R., Bogoshi M., Churchyard G. (2009). The diarylquinoline TMC207 for multidrug-resistant tuberculosis. N. Engl. J. Med..

[9] Osborne R. (2013). First novel anti-tuberculosis drug in 40 years. Nat. Biotechnol..

[10] Makarov V., Manina G., Mikusova K., Mollmann U., Ryabova O., Saint-Joanis B., Dhar N., Pasca M.R., Buroni S., Lucarelli A.P. (2009). Benzothiazinones kill *Mycobacterium tuberculosis* by blocking arabinan synthesis. Science.

[11] Kaneko T., Cooper C., Mdluli K. (2011). Challenges and opportunities in developing novel drugs for TB. Future Med. Chem..

[12] Singh J., Petter R.C., Baillie T.A., Whitty A. (2011). The resurgence of covalent drugs. Nat. Rev. Drug Discov..

[13] Abuhammad A., Fullam E., Lowe E.D., Staunton D., Kawamura A., Westwood I.M., Bhakta S., Garner A.C., Wilson D.L., Seden P.T. (2012). Piperidinols that show anti-tubercular activity as inhibitors of arylamine *N*-acetyltransferase: An essential enzyme for mycobacterial survival inside macrophages. PLoS One.

[14] Bhakta S., Besra G.S., Upton A.M., Parish T., Sholto-Douglas-Vernon C., Gibson K.J., Knutton S., Gordon S., DaSilva R.P., Anderton M.C. (2004). Arylamine *N*-acetyltransferase is required for synthesis of mycolic acids and complex lipids in *Mycobacterium bovis* BCG and represents a novel drug target. J. Exp. Med..

[15] Anderton M.C., Bhakta S., Besra G.S., Jeavons P., Eltis L.D., Sim E. (2006). Characterization of the putative operon containing arylamine *N*-acetyltransferase (*nat*) in *Mycobacterium bovis* BCG. Mol. Microbiol..

[16] Yam K.C., D’Angelo I., Kalscheuer R., Zhu H., Wang J.X., Snieckus V., Ly L.H., Converse P.J., Jacobs W.R., Strynadka N. (2009). Studies of a ring-cleaving dioxygenase illuminate the role of cholesterol metabolism in the pathogenesis of *Mycobacterium tuberculosis*. PLoS Pathog..

[17] Suleyman H., Gul H.I., Asoglu M. (2003). Anti-inflammatory activity of 3-benzoyl-1-methyl-4-phenyl-4-piperidinol hydrochloride. Pharmacol. Res..

[18] Vashishtha S.C., Allen T.M., Halleran S., Szydlowski J., Santos C.L., De Clercq E., Balzarani J., Dimmock J.R. (2001). Cytotoxic and anticancer properties of some 4-aryl-3-arylcarbonyl-1-ethyl-4-piperidinols and related compounds. Pharmazie.

[19] Gul H.I., Calls U., Ozturk Z., Tutar E., Calikiran L. (2007). Evaluation of anticonvulsant activities of bis(3-aryl-3-oxo-propyl) ethylamine hydrochlorides and 4-aryl-3-arylcarbonyl-1-ethyl-4-piperidinol hydrochlorides. Arzneim. Forsch..

[20] Gul H.I., Sahin F., Gul M., Ozturk S., Yerdelen K.O. (2005). Evaluation of antimicrobial activities of several Mannich bases and their derivatives. Arch. Pharm. (Weinheim).

[21] Jeney E., Zsolnai T. (1956). Studies in search of new tuberculostatic drugs. I. Hydrazine derivatives, carbolic acid, phenols, quaternary ammonium compounds and their intermediaries. Zentralbl. Bakteriol. Orig..

[22] Sloan K.B., Koch S.A.M., Siver K.G. (1984). Mannich base derivatives of theophylline and 5-fluorouracil: Syntheses, properties and topical delivery characteristics. Int. J. Pharm..

[23] Brooke E.W., Davies S.G., Mulvaney A.W., Pompeo F., Sim E., Vickers R.J. (2003). An approach to identifying novel substrates of bacterial arylamine *N*-acetyltransferases. Bioorg. Med. Chem..

[24] Westwood I., Bhakta S., Russell A., Fullam E., Anderton M., Kawamura A., Mulvaney A., Vickers R., Bhowruth V., Besra G. (2010). Identification of aryalmine *N*-acetyltransferase inhibitors as an approach towards novel anti-tuberculars. Protein Cell.

[25] Westwood I.M., Kawamura A., Russell A.J., Sandy J., Davies S.G., Sim E. (2011). Novel small-molecule inhibitors of arylamine *N*-acetyltransferases: Drug discovery by high-throughput screening. Comb. Chem. High Throughput Screen..

[26] Fullam E. (2007). Arylamine *N*-Acetyltransferase of *Mycobacteria*. Ph.D. Thesis.

[27] Abuhammad A.M., Lowe E.D., Fullam E., Noble M., Garman E.F., Sim E. (2010). Probing the architecture of the *Mycobacterium marinum* arylamine *N*-acetyltransferase active site. Protein Cell.

[28] Kitz R., Wilson I.B. (1962). Esters of methanesulfonic acid as irreversible inhibitors of acetylcholinesterase. J. Biol. Chem..

[29] PerkinElmer. http://www.cambridgesoft.com.

[30] Fullam E., Kawamura A., Wilkinson H., Abuhammad A., Westwood I., Sim E. (2009). Comparison of the arylamine *N*-acetyltransferase from *Mycobacterium marinum* and *Mycobacterium tuberculosis*. Protein J..

[31] Abuhammad A. (2013). Arylamine *N*-acetyltransferases from Mycobacteria: Investigations of a Potential Target for Anti-Tubercular Therapy. Ph.D. Thesis.

[32] Fullam E., Westwood I.M., Anderton M.C., Lowe E.D., Sim E., Noble M.E. (2008). Divergence of cofactor recognition across evolution: Coenzyme a binding in a prokaryotic arylamine *N*-acetyltransferase. J. Mol. Biol..

[33] Pluvinage B., Sierra-Gallay I.L., Kubiak X., Xu X., Dairou J., Dupret J.M., Rodrigues-Lima F. (2011). The *Bacillus anthracis* arylamine *N*-acetyltransferase ((BACAN)NAT1) that inactivates sulfamethoxazole, reveals unusual structural features compared with the other NAT isoenzymes. FEBS Lett..

[34] Abuhammad A., Lowe E.D., McDonough M.A., Stewart P.D.S., Kolek S.A., Sim E., Garman E.F. (2013). Structure of arylamine *N*-acetyltransferase from *M. tuberculosis* determined by cross-seeding with homologous protein from *M. marinum*: Triumph over adversity. Acta Crystallogr. D.

[35] Ballester P.J., Westwood I., Laurieri N., Sim E., Richards W.G. (2010). Prospective virtual screening with ultrafast shape recognition: The identification of novel inhibitors of arylamine *N*-acetyltransferases. J. R. Soc. Interface.

[36] Armstrong M.S., Finn P.W., Morris G.M., Richards W.G. (2011). Improving the accuracy of ultrafast ligand-based screening: Incorporating lipophilicity into electroshape as an extra dimension. J. Comput. Aided Mol. Des..

[37] Armstrong M.S., Morris G.M., Finn P.W., Sharma R., Moretti L., Cooper R.I., Richards W.G. (2010). Electroshape: Fast molecular similarity calculations incorporating shape, chirality and electrostatics. J. Comput. Aided Mol. Des..

[38] Armstrong M.S., Morris G.M., Finn P.W., Sharma R., Richards W.G. (2009). Molecular similarity including chirality. J. Mol. Graph. Model..

[39] Biovir. http://accelrys.com/products/discovery-studio/.

[40] Cwik A., Fuchs A., Hell Z., Clacens J.-M. (2004). An efficient and environmental-friendly synthesis of 4-hydroxy-arylpiperidines using hydrotalcite catalyst. J. Mol. Catal. A Chem..

[41] Payton M., Auty R., Delgoda R., Everett M., Sim E. (1999). Cloning and characterization of arylamine *N*-acetyltransferase genes from *Mycobacterium smegmatis* and *Mycobacterium tuberculosis*: Increased expression results in isoniazid resistance. J. Bacteriol..

[42] Sinclair J.C., Sandy J., Delgoda R., Sim E., Noble M.E. (2000). Structure of arylamine *N*-acetyltransferase reveals a catalytic triad. Nat. Struct. Biol..

[43] Westwood I. (2005). Structure and Activity of Arylamine N-Acetyltransferase form Pseudomonas aeruginosa.

[44] Abuhammad A., Lack N., Schweichler J., Staunton D., Sim R.B., Sim E. (2011). Improvement of the expression and purification of *Mycobacterium tuberculosis* arylamine *N*-acetyltransferase (TBNAT) a potential target for novel anti-tubercular agents. Protein Expr. Purif..

[45] Kawamura A., Graham J., Mushtaq A., Tsiftsoglou S.A., Vath G.M., Hanna P.E., Wagner C.R., Sim E. (2005). Eukaryotic arylamine *N*-acetyltransferase. Investigation of substrate specificity by high-throughput screening. Biochem. Pharmacol..

[46] Wang W., Zhang C., Marimuthu A., Krupka H.I., Tabrizizad M., Shelloe R., Mehra U., Eng K., Nguyen H., Settachatgul C. (2005). The crystal structures of human steroidogenic factor-1 and liver receptor homologue-1. Proc. Natl. Acad. Sci. USA.

[47] Evangelopoulos D., Bhakta S., Gillespie S.H., McHugh T.D. (2010). Rapid methods for testing inhibitors of mycobacterial growth. Antibiotic Resistance Protocols.

[48] Russell A.J., Westwood I.M., Crawford M.H., Robinson J., Kawamura A., Redfield C., Laurieri N., Lowe E.D., Davies S.G., Sim E. (2009). Selective small molecule inhibitors of the potential breast cancer marker, human arylamine *N*-acetyltransferase 1, and its murine homologue, mouse arylamine *N*-acetyltransferase 2. Bioorg. Med. Chem..

[49] Yajko D., Madej J., Lancaster M., Sanders C., Cawthon V., Gee B., Babst A., Hadley W. (1995). Colorimetric method for determining mics of antimicrobial agents for *Mycobacterium tuberculosis*. J. Clin. Microbiol..

[50] Franzblau S.G., Witzig R.S., McLaughlin J.C., Torres P., Madico G., Hernandez A., Degnan M.T., Cook M.B., Quenzer V.K., Ferguson R.M. (1998). Rapid, low-technology mic determination with clinical *Mycobacterium tuberculosis* isolates by using the microplate alamar blue assay. J. Clin. Microbiol..

[51] Ebejer J.-P., Morris G.M., Deane C.M. (2012). Freely available conformer generation methods: How good are they?. J. Chem. Inf. Model..

