# Supplementary material for: Exploration of Piperidinols as Potential Antitubercular Agents
Source: Molecules. 2014 Oct 10;19(10):16274–90. doi: 10.3390/molecules191016274 (PMC6271891; doi:10.3390/molecules191016274)
Supplement: Supplementary File 1 [file molecules-19-16274-s001.pdf]

Supplementary Materials

Figure S1. The time-dependent inhibition of the MMNAT by the piperidinols (**A**) compounds **1**, **3**, **4**, **6** at 11.9 µM concentration (**B**) **5**, **7** at 5.9 µM concentration and (**C**) **8**, **9** (11.9 µM).

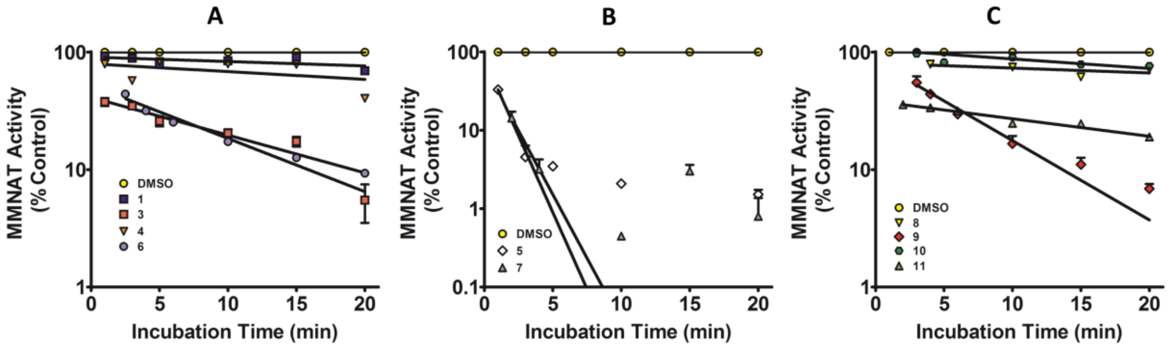

Semi-logarithmic plots showing the time-dependent inactivation of MMNAT by (A) compounds **1**, **3**, **4**, **6** at 11.9 µM concentration (B) **5**, **7** at 5.9 µM concentration and (C) **8**, **9** (11.9 µM), **10** (23.8 µM) and **11** (5.9 µM). The enzyme activity was measured using the protocol described in [1]. The results are presented as the mean ± S.D. of triplicate measurements. The error bars are within the symbols. The residual activity is shown as a percentage of a control. The data were fitted against the incubation time using the Semilog line (X is linear, Y is Log) module of GraphPad Prism 5.0. The slope of each line is equivalent to ($-k_{obs}$/2.303) at each inhibitor concentration.

Figure S2. Correlation between $t_{1/2}$ with the molar critical volume and cLogP of the piperidinols. $t_{1/2}$, the critical volume and cLogP values were obtained as described in Table 1. (**A**) No correlation was found between $t_{1/2}$ and critical volume with $r^2 = 0.04$ and $p = 0.53$. (**B**) No correlation was found between $t_{1/2}$ and cLogP, with $r^2 = 0.07$ and $p = 0.43$. The correlation coefficient (squared, r^2) and p value were calculated by the Correlation module of GraphPad Prism 5.0.

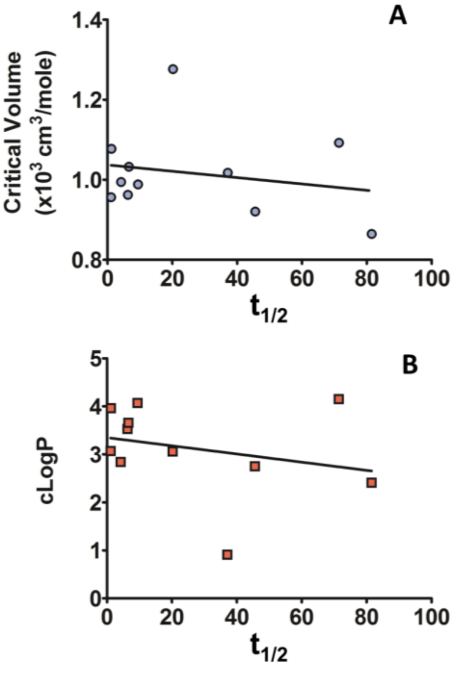

Figure S3. Electron density in active site of MMNAT.

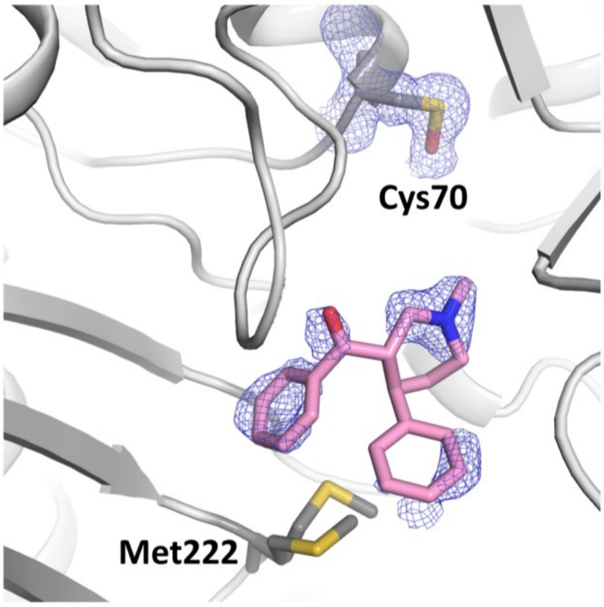

The excess 2Fo–Fc electron (0.9 σ) observed in the MMNAT-**1** complex (PDB entry 4c5p; [2]) obtained by soaking a crystal of MMNAT in 1 mM compound **1** dissolved in the mother liquor [2]. A model of compound **1** (in pink) docked in this electron density using COOT [3] is also shown. The electron density corresponds to the oxidation of the cysteine residue Cys70 into sulfenic acid (CSO70, occupancy 75%). The key amino acid residues are labelled. The figure was prepared using PyMOL [4].

Figure S4. The 3D-shape of compound **1** in comparison with the shapes of compounds **15** and **16**. The mesh view of the 3D-shape in form of Van der Waals surface of the query **1** conformer used in the *in silico* screen, the active hits **15** and **16**. The figure was prepared by using the DS Visualizer 3.1.

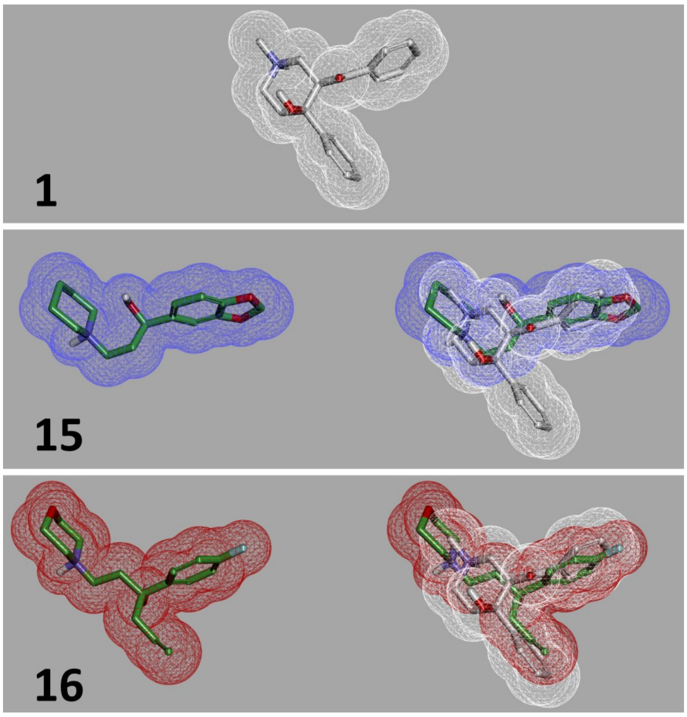

Figure S5. The Alamar blue assay of *M. bovis* BCG with **15** and INH as a control. The inhibition of *M. bovis* growth in liquid media was assessed from the colour change of the Alamar blue at 24 h of incubation as shown. A red colour indicates growth/resistance, and blue indicates no growth/sensitivity. The results are compared to those obtain with isoniazid (INH). MICs were determined visually. Note different scale.

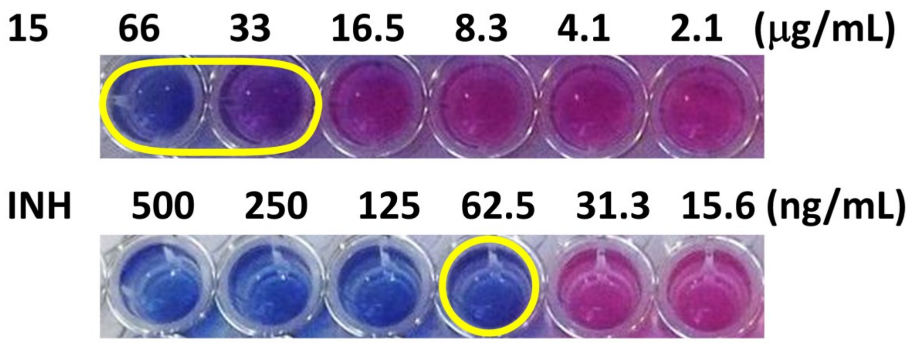

Figure S6. ^{1}H-NMR (400 MHz $CDCl_3$ Bruker AV400 spectrometer) of compound **1**.

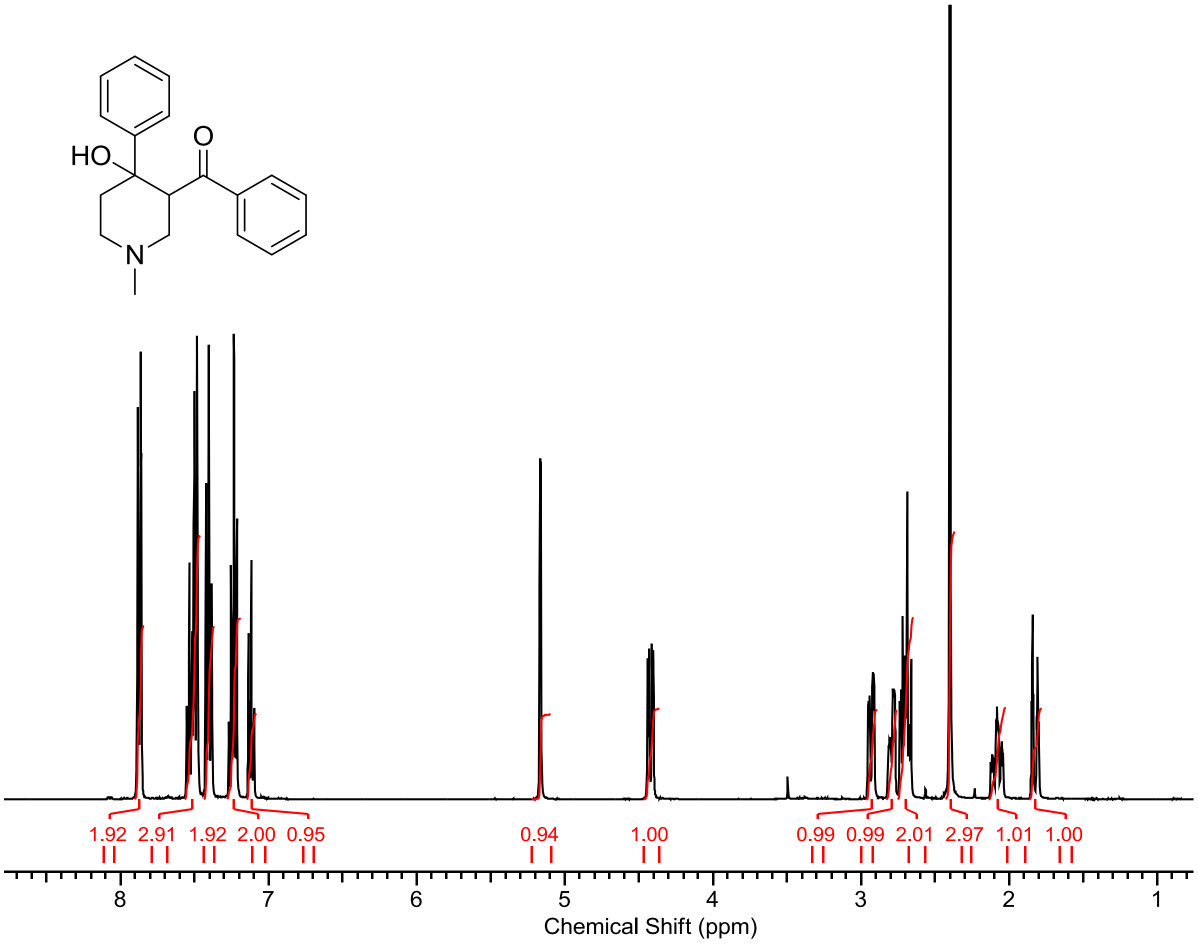

Figure S7. ^{13}C-NMR(75 MHz $CDCl_3$ Bruker DPX300 spectrometer) of of compound **2**.

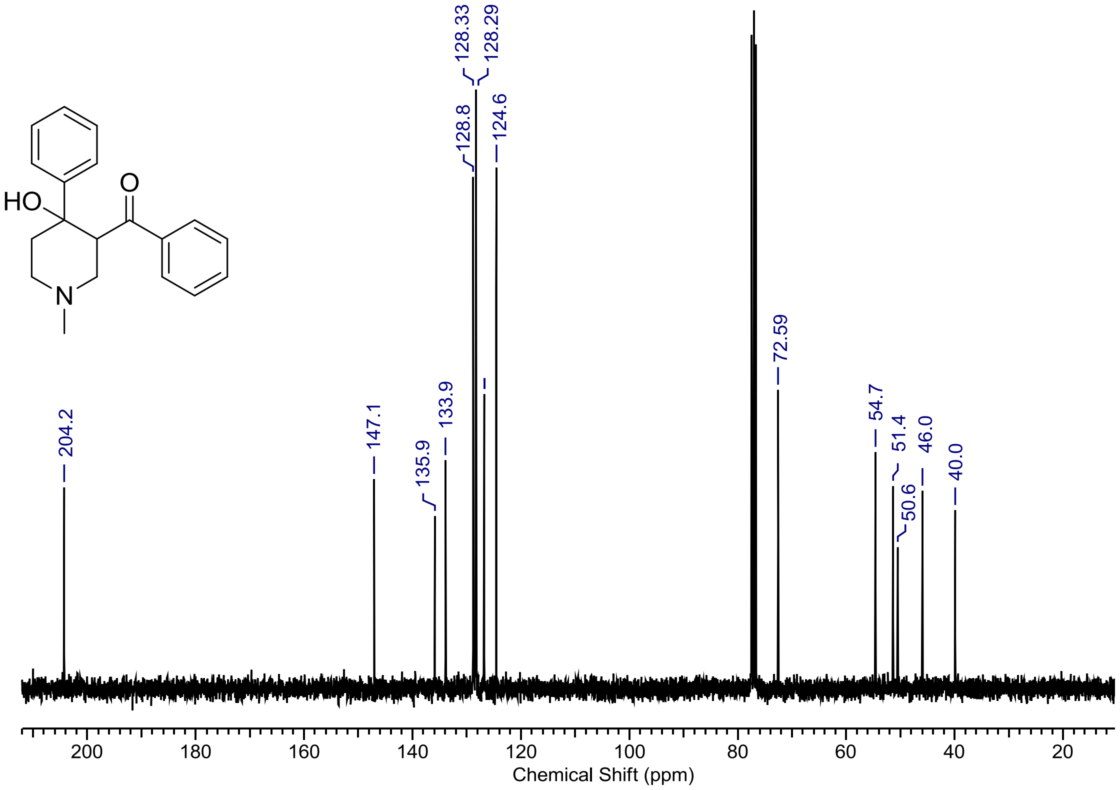

Figure S8. ^{1}H-NMR(400 MHz $CDCl_3$ Bruker AV400 spectrometer) of compound **3**.

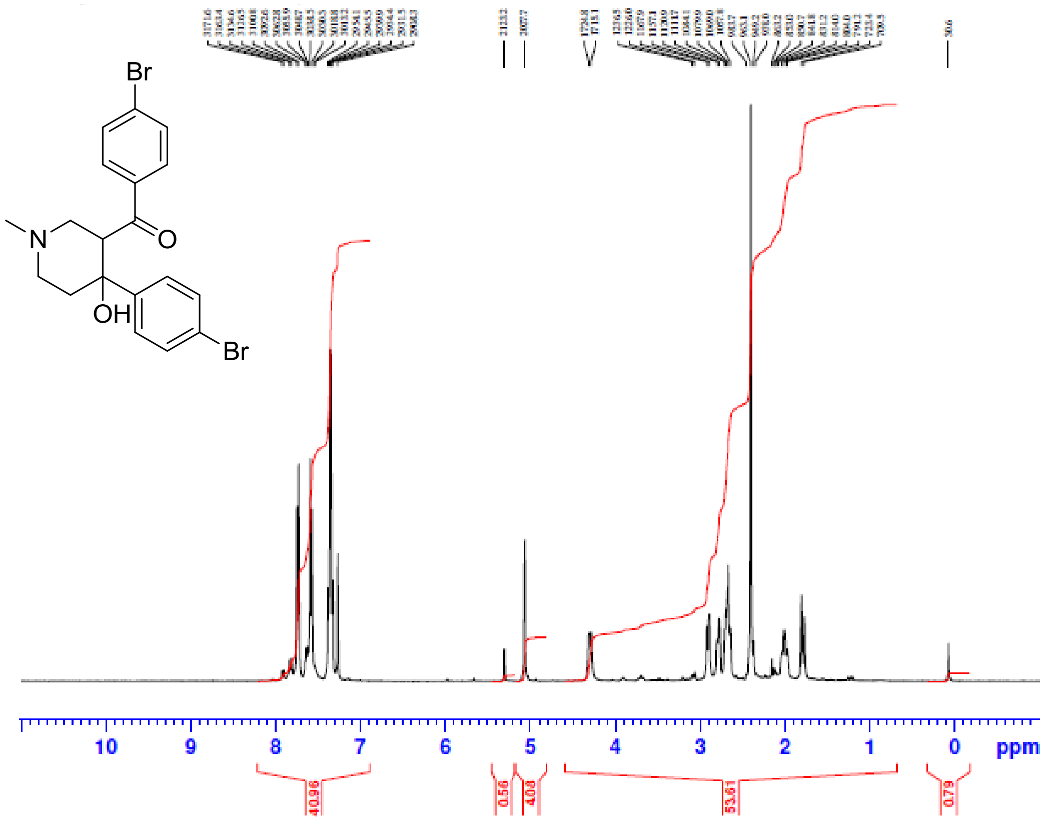

Table S1. The chemical scaffolds of the tested 3D-shape hits with their *in silico* scores and their experimental activities against TBNAT and MMNAT [a].

Code	Structure	ElectroShape score	*In silico* hit rank	TBNAT		MMNAT	
				% Inhibition	IC_{50} (μM)	% Inhibition	IC_{50} (μM)
1	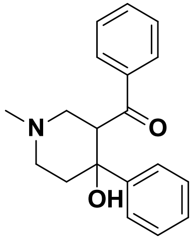	0.995	1	101 ± 2	7.7 ± 0.9	105 ± 1	1.3 ± 0.0
12	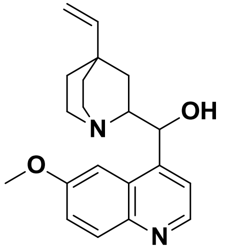	0.949	8	16 ± 5	ND	35 ± 3	ND
13	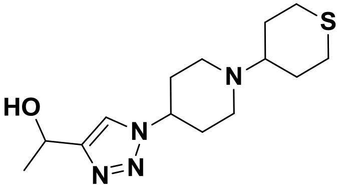	0.935	46	−17 ± 1	ND	23 ± 2	ND
14	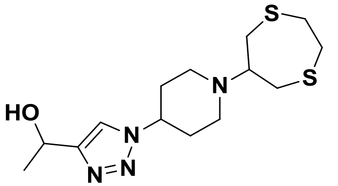	0.931	68	−21 ± 3	ND	31 ± 5	ND
15	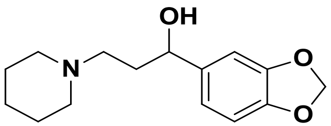	0.964	5	86 ± 2	20 ± 3	103 ± 3	1.0 ± 0.1

Table S1. *Cont.*

Code	Structure	ElectroShape score	*In silico* hit rank	TBNAT		MMNAT	
				% Inhibition	IC_{50} (µM)	% Inhibition	IC_{50} (µM)
16	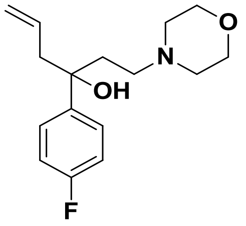	0.930	70	−14 ± 3	ND	99 ± 1	6.4 ± 0.6
17	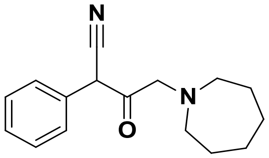	0.937	76	−35 ± 1	ND	12 ± 2	ND
18	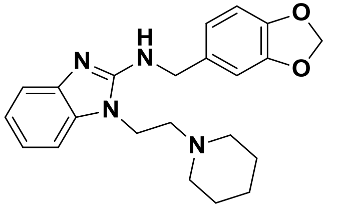	0.923	91	14 ± 6	ND	16 ± 2	ND
19	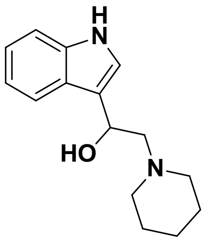	0.931	66	11 ± 6	ND	10 ± 1	ND

Table S1. *Cont.*

Code	Structure	ElectroShape score	*In silico* hit rank	TBNAT		MMNAT	
				% Inhibition	IC_{50} (µM)	% Inhibition	IC_{50} (µM)
20	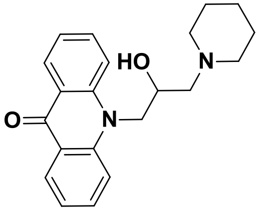	0.923	90	9 ± 7	ND	21 ± 4	ND
21	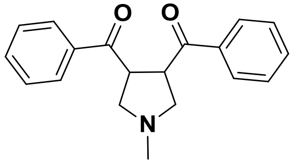	0.928	76	−15 ± 4	ND	3 ± 3	ND
22	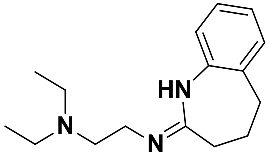	0.921	100	5 ± 1	ND	57 ± 1	ND
23	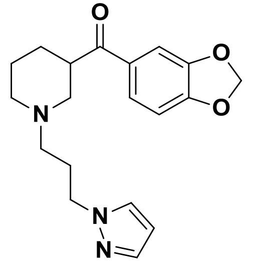	0.940	24	**−13** ± 1	ND	46 ± 2	ND

[a] Compound 1 was used as a query molecule. The rank of the hits, according to the ElectroShape scores, are shown alongside the corresponding scores. The NAT activity was measured by the NAT-inhibition assay using 150 µM HLZ and 120 µM Ac-CoA as substrates as described Methods. The percentage of enzyme inhibition was measured in the presence of 50 µM inhibitor and compared to the un-inhibited control. The IC_{50} values were determined by measuring the enzyme activity in the presence of variable concentrations of each inhibitor (0–250 µM) and compared to the un-inhibited control. The results are presented as the mean ± S.D. of triplicate measurements. ND is not determined. Inhibition curves obtained by non-linear fitting of the % inhibition and the inhibitor concentration (µM) using the Log(inhibitor) *vs.* response module of GraphPad Prism 5.0.

References

1. Abuhammad, A.; Fullam, E.; Lowe, E.D.; Staunton, D.; Kawamura, A.; Westwood, I.M.; Bhakta, S.; Garner, A.C.; Wilson, D.L.; Seden, P.T.; *et al.* Piperidinols that show anti-tubercular activity as inhibitors of arylamine N-acetyltransferase: An essential enzyme for mycobacterial survival inside macrophages. *PLoS One* **2012**, *7*, e52790.
2. Abuhammad, A. Arylamine *N*-Acetyltransferases from Mycobacteria: Investigations of a Potential Target for Anti-Tubercular Therapy. Ph.D. Thesis, University of Oxford, Oxford, UK, April 2013.
3. Emsley, P.; Lohkamp, B.; Scott, W.G.; Cowtan, K. Features and development of Coot. *Acta Crystallogr., Sect. D: Biol. Crystallogr.* **2010**, *66*, 486–501.
4. Schrodinger, LLC. *The PyMOL Molecular Graphics System*, version 1.3r1; Schrodinger, LLC: Camberley, UK, 2010.
